# The effect of psychological treatment on repetitive negative thinking in youth depression and anxiety: a meta-analysis and meta-regression

**DOI:** 10.1017/S0033291722003373

**Published:** 2023-01

**Authors:** Imogen H. Bell, Wolfgang Marx, Katherine Nguyen, Sally Grace, John Gleeson, Mario Alvarez-Jimenez

**Affiliations:** 1 Orygen, Melbourne, Australia; 2 Centre for Youth Mental Health, University of Melbourne, Melbourne, Australia; 3 Deakin University, IMPACT – the Institute for Mental and Physical Health and Clinical Translation, Food & Mood Centre, School of Medicine, Barwon Health, Geelong, Australia; 4 Healthy Brain and Mind Research Centre and School of Behavioural and Health Sciences, Australian Catholic University, Melbourne, Victoria, Australia

**Keywords:** Anxiety psychological treatment, depression, repetitive negative thinking, rumination, transdiagnostic, worry, youth mental health

## Abstract

**Background:**

Depression and anxiety are prevalent in youth populations and typically emerge during adolescence. Repetitive negative thinking (RNT) is a putative transdiagnostic mechanism with consistent associations with depression and anxiety. Targeting transdiagnostic processes like RNT for youth depression and anxiety may offer more targeted, personalised and effective treatment.

**Methods:**

A meta-analysis was conducted to examine the effect of psychological treatments on RNT, depression and anxiety symptoms in young people with depression or anxiety, and a meta-regression to examine relationships between outcomes.

**Results:**

Twenty-eight randomised controlled trials examining 17 different psychological interventions were included. Effect sizes were small to moderate across all outcomes (Hedge's g depression = −0.47, CI −0.77 to −0.17; anxiety = −0.42, CI −0.65 to −0.20; RNT = −0.45, CI −0.67 to −0.23). RNT-focused and non-RNT focused approaches had comparable effects; however, those focusing on modifying the process of RNT had significantly larger effects on RNT than those focusing on modifying negative thought content. Meta-regression revealed a significant relationship between RNT and depression outcomes only across all intervention types and with both depression and anxiety for RNT focused interventions only.

**Conclusion:**

Consistent with findings in adults, this review provides evidence that reducing RNT with psychological treatment is associated with improvements in depression and anxiety in youth. Targeting RNT specifically may not lead to better outcomes compared to general approaches; however, focusing on modifying the process of RNT may be more effective than targeting content. Further research is needed to determine causal pathways.

Anxiety and depressive disorders are highly debilitating and amongst the most common psychological disorders worldwide (Kessler *et al*., 2005, 2007; Murray *et al*., 2020). Occurring at a time of peak development, 75% of mental disorders emerge before the age of 25 (Kessler *et al*., 2007), presenting lasting social, psychological and functional consequences (Bruce *et al*., 2005; Clayborne, Varin, & Colman, 2019; Gibb, Fergusson, & Horwood, 2010). The prevalence of mental disorders amongst adolescents is estimated to be 13.4% and represent the highest burden of disease, with suicide the leading cause of death in this age group (Gore *et al*., 2011; Polanczyk, Salum, Sugaya, Caye, & Rohde, 2015).

A large body of research supports the efficacy of psychological interventions for the treatment of emotional disorders in youth (Weisz *et al*., 2017). However, overall treatment effects are modest and evidence for sustained maintenance effects is lacking, particularly for depressive disorders. Indeed, recurrence rates over five years are as high as 83% in depression and 58% for anxiety disorders (Bruce *et al*., 2005).

In pursuit of advancing the effectiveness of psychological treatments, there have been recent calls to move away from disorder-specific approaches towards a focus on the ‘transdiagnostic’ mechanisms which underpin a range of psychological disorders (Barlow, Allen, & Choate, 2016; Bullis, Boettcher, Sauer-Zavala, Farchione, & Barlow, 2019; Dalgleish, Black, Johnston, & Bevan, 2020; Ehrenreich-May & Chu, 2013; McEvoy, Nathan, & Norton, 2009). Proponents have pointed to the high rates of comorbidity amongst mental disorders and considerable symptom overlap, suggesting shared underlying aetiology (Dalgleish *et al*., 2020). Estimated comorbidity rates amongst emotional disorders are 55–76%, and 57–81% between emotional disorders and other axis I conditions (Brown, Campbell, Lehman, Grisham, & Mancill, 2001; Lamers *et al*., 2011). Further, similar treatments yield similar effect sizes across disorders, highlighting issues with specificity (Dalgleish *et al*., 2020; Weisz *et al*., 2017). Alternatively, targeting underlying psychopathological process that is common across disorders in treatment may be more effective and efficient (Barlow *et al*., 2016; McEvoy *et al*., 2009).

A number of candidate transdiagnostic mechanisms have been identified in studies that typically isolate and examine one or more pathological processes in samples with mixed diagnoses (Dalgleish *et al*., 2020; Ehrenreich-May & Chu, 2013). Amongst emotional disorders, a widely studied transdiagnostic mechanisms with consistent linkages to depression and anxiety is repetitive negative thinking (RNT; Ehring & Watkins, 2008). RNT is defined as a pattern of thinking that is repetitive, passive or difficult to control, and focused on negative content. For anxiety disorders, RNT is typically conceptualised as worry: verbal strings of negative thought relating to future threats (e.g. worry about failing an upcoming test; Olatunji, Wolitzky-Taylor, Sawchuk, & Ciesielski, 2010). For depressive disorders, RNT is generally associated with rumination: fixation of thoughts on negative events of the past or on one's present symptoms (e.g. excessively thinking about a prior regret; Nolen-Hoeksema, Wisco, & Lyubomirsky, 2008). Although worry and rumination have traditionally been regarded as distinct constructs, recent research indicates that they share common processes and overlap in their relationships to various psychopathologies (McEvoy, Watson, Watkins, & Nathan, 2013; Spinhoven, Drost, van Hemert, & Penninx, 2015). As such, there is evidence to suggest that although worry and rumination differ in their content, their effects are likely explained by the broader, unitary, transdiagnostic process of RNT (Ehring & Watkins, 2008). In youth populations, RNT has been consistently linked to depression and anxiety (McEvoy *et al*., 2019; Rood, Roelofs, Bögels, & Alloy, 2010). Longitudinal studies have shown that RNT predicts the onset and maintenance of anxiety and depressive disorders in both adult and adolescent populations (Raes, 2012; Spinhoven, van Hemert, & Penninx, 2018b; Wilkinson, Croudace, & Goodyer, 2013; Young & Dietrich, 2015), and experimental studies have shown that inducing RNT directly leads to increased negative emotional states (McLaughlin, Borkovec, & Sibrava, 2007).

A number of therapeutic approaches have been developed that focus on reducing RNT specifically (see Topper, Emmelkamp, & Ehring, 2010). In accordance with varying conceptualisations of RNT, different techniques have emerged for targeting the modification of either the content of the negative thought or the process of repeatedly thinking about negative thoughts. The distinction between these approaches aligns with the so-called *second* and *third waves of* cognitive behavioural therapy (CBT; Hayes, 2004). Second wave approaches, comprising of traditional CBT and associated strategies such as cognitive restructuring, focus primarily on modifying underlying cognitions that maintain emotional problems. For example, examining evidence for and against the likelihood of a future undesirable event happening in the case of worry. Third wave approaches, such as acceptance and commitment therapy (ACT), have focused on adapting awareness of, and relationship to, thoughts and emotions. For example, using mindfulness techniques to allow the thought to come and go without reacting. In the treatment of RNT, content focused interventions (i.e. second wave) have typically distinguished worry from rumination because these forms of RNT differ in content (i.e. past or future focused). In contrast, process focused interventions (i.e. third wave) primarily target the process and functions of RNT as a broader construct irrespective of thought content, using techniques such as mindfulness. Whilst the distinction between these approaches is not clear cut, observing the relative effects of different approaches may offer some insight into the clinical and theoretical significance of distinguishing between dimensions of RNT (Deacon, Fawzy, Lickel, & Wolitzky-Taylor, 2011).

Studies point towards RNT playing a causal role in the development of depression and anxiety in youth, and as such, treatment that reduces RNT may offer flow on effects for the improvement of depression and anxiety outcomes (Topper *et al*., 2010). Two recent meta-analyses in adult populations examined the effect of psychological treatments for anxiety (Monteregge, Tsagkalidou, Cuijpers, & Spinhoven, 2020) and depression (Spinhoven *et al*., 2018a) on RNT. Both reviews found that reductions in RNT correspond with reductions in depression and anxiety outcomes, and that this did not differ depending on whether targeting reduction in RNT was the specific focus of the treatment. However, Spinhoven *et al*. (2018a, 2018b) found that this relationship was most pronounced for treatments that focused on reducing RNT specifically. These findings point towards the clinical significance of reducing RNT for improving depression and anxiety outcomes in adults, however this yet to be investigated in youth populations. Transdiagnostic interventions in youth may address the high rates of comorbidity seen in this population (Garber & Weersing, 2010) and provide more targeted and personalised treatments relevant to young people and clinicians (Weisz, Krumholz, Santucci, Thomassin, & Ng, 2015). In the context of early intervention, treating RNT at early stages may prevent progression to a full-blown emotional disorder in adolescence (McGorry & Nelson, 2016; Shah *et al*., 2020). Making sure that these interventions are developmentally appropriate is important to ensure they are acceptable and effective for the unique needs and experiences of young people. Given the importance of RNT as a treatment target in youth, and with a number of treatments now developed and tested in youth populations, it is timely to review this literature. Therefore, the overall aim of the current review was to examine the effect of psychological treatment on RNT, depression and anxiety in young people with depression and/or anxiety. Examining effect sizes may also inform the theoretical understanding of transdiagnostic models of intervention. Firstly, a key argument underpinning the transdiagnostic theory is that identifying and targeting transdiagnostic processes in treatment will lead to greater improvements across different mental health conditions, and that these improvements are driven by the successful modification of these processes. This theory can be tested by directly comparing treatments which target RNT specifically to those more general approaches which do not employ techniques to reduce RNT as a primary focus. Further, examining the association between RNT, depression and anxiety outcomes can reveal the degree to which changes in these outcomes may be related. Secondly, comparing the relative effect of interventions which primarily target either the process or content of RNT may offer insight into the best treatment approaches and reveal the degree to which the content of the negative thought (i.e. by separating worry from rumination) is critical to the construct of RNT. Comparisons between these approaches have not been investigated in previous reviews. The following questions were the primary focus of this review:

1. What is the effect of psychological treatment on RNT, depression and anxiety in youth, and what is the relationship between these outcomes?
2. Does the effect of these treatments on RNT differ depending on whether RNT is targeted specifically in treatment or not?
3. Does the effect of these treatments on RNT outcomes differ depending on whether the treatment targeted the process or content of RNT?

## Method

The review followed PRISMA guidelines (see online Supplementary; Moher, Liberati, Tetzlaff, & Altman, 2009) and was prospectively registered (PROSPERO: CRD42020196415).

### Search strategy and selection criteria

The Orygen Evidence Finder was used to identify relevant English language studies as a first stage (for more details about this database and the search approach see online Supplementary, and De Silva, Bailey, Parker, Montague, & Hetrick, 2018; Hetrick, Parker, Callahan, & Purcell, 2010). The database is populated annually using systematic searches of the MEDLINE, PsycINFO, EMBASE databases, and the Cochrane Database of Systematic Reviews. Screening of 380 000 records published between 1980 and June 2020 was conducted, from which 4759 studies were included and categorisation within the database. Each record retrieved was screened independently by two reviewers against the eligibility criteria, first by title and abstract and second by full text. Discrepancies between reviewers were resolved via discussion with a third reviewer. For the current review, a third stage involved screening all full text articles that had been categorized in the database as: (1) randomised controlled trials; (2) psychological interventions; and (3) sample meeting criteria for any depressive or anxiety disorder, or meeting clinical cut-off criteria for depression and/or anxiety.

Full text studies were then screened according to the following inclusion criteria by two independent reviewers (SG and KN), with conflicts resolved by third party consensus (IB): (1) sample mean age between 14–24 years (corresponding to the peak age range of mental disorder onset; Solmi *et al*., 2022); (2) sample meets criteria for any depressive or anxiety disorder, or clinically elevated levels of depression and/or anxiety according to standardised measures (either as an inclusion criteria or mean scores of the whole sample at baseline meeting cut-off criteria for clinical levels of depression and/or anxiety); (3) trial investigated any psychological intervention; (3) design included a control group and allocation was randomised; (4) included outcomes using any validated measure of RNT (including rumination and worry) and depression or anxiety. Studies not meeting these criteria were excluded. Studies were eligible for the meta-analysis if they reported pre and post intervention means and standard deviations for each outcome measure (RNT, depression and/or anxiety) for each group.

### Data extraction and analysis

Two independent reviewers (KN and SG/IB) extracted the following data from included studies: basic study and sample information, details of the intervention type, delivery medium and setting, trial design and outcome measures, and group means and standard deviations for each outcome measure pre and post intervention. To facilitate subgroup comparisons, trials (or intervention arms within trials) were categorised as RNT and non-RNT focused, and process or content focused, based on whether this was specifically stated in the study or via consensus amongst independent reviewers (KN, SG, IHB), in reference to relevant literature (Hayes, 2004; Topper *et al*. 2010), with conflicts resolved via discussion with senior authors (MAJ, JG). The coding of RNT focused and non-RNT focused was operationalised by whether or not the intervention explicitly targeted either the process of repeated attention towards negative thoughts (e.g. mindfulness or acceptance approaches) or the content of negative biases that perpetuated repeated focus on negative thoughts (e.g. attention bias modification, ABM).Comprehensive Meta-Analysis software (version 3.3) was used to calculate pooled standardised effect sizes (hedge's g) and their 95% CIs for each comparison (0.2 = small; 0.5 = moderate; 0.8 = large; Cohen, 2013). Effect sizes were calculated for each comparison between an active intervention and control comparison using group means and standard deviation at the post assessment timepoint. For studies with multiple intervention arms, effect sizes of the separate intervention groups were combined to produce a pooled effect size. For studies with more than one outcome measure per construct, effect sizes were pooled across outcomes to yield a single effect size for each measure. A random-effects model was used in all analyses due to heterogeneity, which was assessed using *I^2^* (low-25%, medium-50%, high-75%) and a *Q* statistic. Duval and Tweedie's (2000) trim and fill procedure involving inspection of the funnel plot was followed to examine publication bias, with an Egger's test for asymmetry. Subgroup analyses were conducted to compare effect sizes for RNT across different subgroups. Subgroups included type of intervention (RNT or non-RNT focused, and process or content-focused), format (group, individual or self-guided), number of sessions (more or less than eight, or continuous), delivery medium (group, individual, digital or self-guided), clinical group (elevated depression or anxiety, depressive disorder or anxiety disorder), age (older or younger than 18 years), recruitment setting, and study quality (high or low quality). As conducted in previous meta-analyses in this field (Monteregge *et al*., 2020; Spinhoven *et al*., 2018a, 2018b), to examine the relationship between depression, anxiety and RNT outcomes, a meta-regression was conducted with post-intervention RNT as the predictor and depression and anxiety as the outcome variable.

### Risk of bias

Two independent reviewers (KN and IB) used the Cochrane Risk of Bias tool (Higgins *et al*., 2011) to assess study quality across five domains: allocation concealment, random sequence generation, masking of assessors, incomplete outcome data, and selective reporting. Studies with high-risk scores in any domain, or some concerns in two or more of the five domains, were rated as high risk. Studies scoring as low risk in at least four domains and maximum some concerns in only one domain were rated as low risk.

## Results

**Fig. 1.**
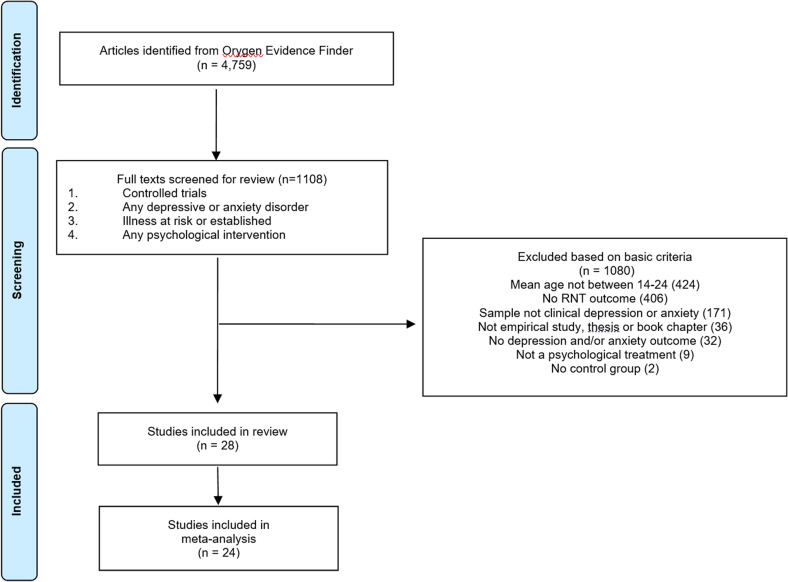
PRISMA flow diagram.

Of the 1108 full text articles screened, 28 met inclusion criteria (see Fig. 1). A list of all included studies and summary of key study characteristics are included in the online Supplementary materials.

### Study characteristics

A total sample of 2498 participants were included across the 28 studies with a mean age of 19.70 (s.d. = 3.71) and average of 76% females within the sample. Three trials included samples of participants with a diagnosed anxiety disorder, three with a diagnosed depressive disorder, 14 with clinically elevated levels of anxiety, five with clinically elevated levels of depression, and three with clinically elevated levels of depression and anxiety. Four recruited a community sample, one through an outpatient clinic, and 23 through a school or university.

Six studies examined multiple interventions compared to a control group, resulting in 17 unique interventions being investigated. These broadly fit into interventions which targeted RNT directly (17 interventions; 20 trials) and broader interventions that did not target RNT directly (nine interventions; eight trials). The most common intervention was ABM and forms of cognitive behavioural therapy. Ten interventions were delivered by a clinician, 13 were self-guided using a computer, 5 delivered in a group format, and 5 were online interventions or self-guided book. Sessions ranged from one (most commonly computerised self-guided interventions delivering a form of ABM) to 28, with a mode of one and median of six.

### Effects on depression, anxiety and RNT

**Fig. 2.**
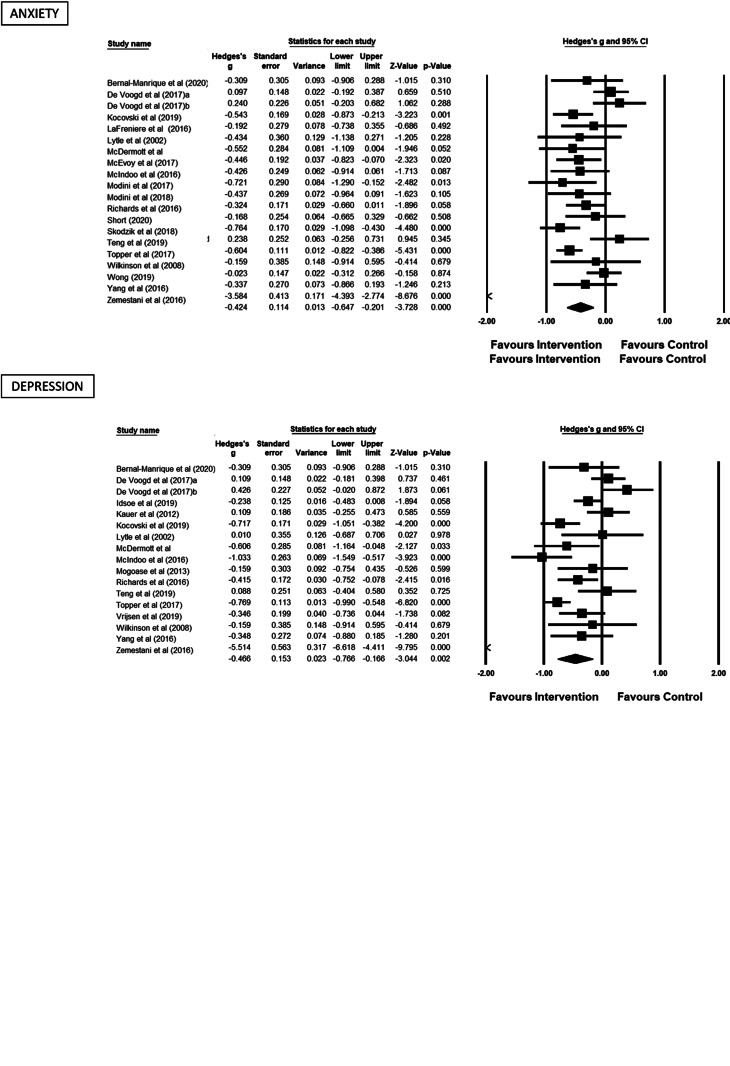
Forrest plots for RNT, depression and anxiety outcomes.

Meta-analysis was conducted on 24 of the 28 studies, with 4 being excluded due to incomplete reporting of post intervention means and standard deviations (Grol *et al*., 2018; McIntosh, 2013; Norr *et al.,* 2014; Sass, Evans, Xiong, Mirghassemi, & Tran, 2017). The pooled effect sizes and 95% confidence intervals of the 31 comparisons between active treatment v. control groups at the post intervention timepoint for each outcome measure is shown in Table 1. All three outcomes showed improvement favouring the treatment group in a similar small to moderate range, which was statistically significant. Forrest plots for each of the three outcomes are presented in Fig. 2.

**Table 1.** Pooled Hedge's *g* effect sizes for depression, anxiety and RNT outcomes

	Number of studies	Hedge's *g* (95% CI)	*p* value	Heterogeneity
Pooled effects				
Depression	17	−0.47 (−0.77 to −0.17)	<0.001	*I^2^* = 88.82%, *Q* = 143.07, *p* < 0.001
Anxiety	20	−0.42 (−0.65 to −0.20)	<0.001	*I^2^* = 81.67%, *Q* = 103.67, *p* < 0.001
RNT	24	−0.45 (−0.0.67 to −0.23)	<0.001	*I^2^* = 84.81%, *Q* = 151.43, *p* < 0.001

Heterogeneity was high and the *Q* value was statistically significant for each analysis (*p* < 0.05), indicating that the effect sizes for each outcome varied between studies. Funnel plots (see online Supplementary) showed evidence of asymmetry in the studies; however, Egger's test was not significant for any of the three analyses (RNT: *t*(22) = 1.16, *p* = 0.12; depression: *t*(15) = 0.79, *p* = 0.44; anxiety: *t*(18) = 0.83, *p* = 0.42). The primary effect sizes were unaffected by a random effects trim-and-fill analysis. In a sensitivity analysis, one study was removed from each analysis due to very large effect sizes (Zemestani *et al*., 2016). As removal did not affect the results, they were retained in the analyses. Furthermore, the results of *leave one out* sensitivity analyses for each outcome demonstrate that no individual study significantly affected the pooled effect size.

### Comparison of effects across subgroups

Subgroup analyses were conducted to compare pooled effect sizes for RNT across different types of studies. Analyses revealed that the effect size of RNT (*n* = 17) and non-RNT (*n* = 9) focused interventions did not significantly differ, although this approached significance (*p* = 0.06) with the direction very slightly favouring RNT focused interventions. However, heterogeneity was still high, suggesting variable effect sizes within these subgroups. Interventions which were primarily focused on modifying the process of RNT (*n* = 10), such as mindfulness and ACT, were compared to interventions aiming to modify the content of negative thoughts (*n* = 14) using traditional second wave approaches such as cognitive behavioural therapy. Analyses revealed that interventions which primarily targeted the process of RNT had significantly larger effects than content focused interventions (*p* = 0.01). Whilst heterogeneity was lower in content focused interventions, variability in effect sizes was still high amongst process focused interventions, suggesting that this effect may vary depending on the intervention type. To further interrogate this effect, sensitivity analyses were conducted whereby this subgroup comparison was re-run with trials of ABM (which may theoretically fit either category and represented the largest number of studies in this analysis) re-coded into the opposite subgroup. The significant effect favouring process focused interventions remained (*p* = 0.05; see online Supplementary S6). Other significant differences were found for interventions delivered via group (*n* = 4), individual clinician (*n* = 5) or self-guided (*n* = 16), with group interventions yielding the largest effect size (*p* = 0.02). No other statistically significant differences were found (Table 2).

**Table 2.** Pooled Hedge's *g* effect sizes for RNT outcomes in each subgroup

	Number of studies	Hedge's *g* (95% CI)	*p* value	Heterogeneity (*I*^2^)
Subgroups				
RNT v non-RNT				
RNT focused interventions	17	−0.50 (−0.78 to −0.22)	0.06	84.81
Non-RNT focused interventions	9	−0.52 (−0.87 to −0.17)		82.07
Content v process				
Content focused intervention	14	−0.13 (−0.28 to 0.01)	0.01	38.28
Process focused intervention	10	−0.85 (−1.29 to −0.41)		89.45
Intervention format				
Group	4	−1.35 (−2.29 to −0.41)	0.02	95.16
Individual	5	−0.56 (−1.11 to −0.01)		81.08
Digital or self-guided	16	−0.24 (−0.44 to −0.04)		72.12
Number of sessions				
Less than 8 sessions	16	−0.58 (−0.89 to −0.27)	0.12	86.80
More than 8 sessions	4	−0.06 (−0.45 to 0.33)		76.14
Continuous	4	−0.35 (−0.70 to 0.01)		64.46
Clinical group				
Elevated depression and/or anxiety	18	−0.31 (−0.52 to −0.10)	0.30	78.99
Clinical depression	2	−1.53 (−4.67 to 1.61)		98.23
Clinical anxiety	4	−0.64 (−1.07 to −0.21)		57.31
Sample age				
Younger than 18 years	9	−0.27 (−0.58 to 0.04)	0.20	84.97
18 years or older	15	−0.56 (−0.88 to −0.24)		84.32
Recruitment context				
Community	4	−0.30 (−0.60 to 0.00)	0.42	66.48
University/school	9	−0.47 (−0.75 to −0.19)		87.14
Clinical setting	1	−0.93 (−1.73 to −0.14)		0
Study quality				
Low quality	7	−0.39 (−0.54 to −0.25)	0.49	90.82
High quality	17	−0.39 (−0.63 to −0.15		81.20

### Relationship between RNT, depression and anxiety outcomes

Following the approach used in Monteregge *et al*. (2020), meta-regression was conducted to examine the relationship between outcomes at the post assessment timepoint. Results indicated a significant, positive association between RNT and depression outcomes (*p* = 0.04), however the association between anxiety and RNT outcomes only approached significance (*p* = 0.07). In subgroups of studies that involved RNT focused interventions and non-RNT focused interventions, results showed that whilst the relationships between RNT and depression and anxiety outcomes were all significant and positive, the strength was greater for RNT focused interventions. A further sensitivity analysis was performed to remove one outlier study (Zemestani, Davoodi, Honarmand, Zargar, & Ottaviani, 2016), with results showing that the association between RNT and depression and anxiety outcomes remained unchanged for RNT focused interventions, however the associations for non-RNT focused interventions were no longer significant. This finding would suggest that the association between RNT, depression and anxiety is stronger for groups of interventions that target RNT directly in treatment, when taking into account the outlier study (Table 3).

**Table 3.** Results of meta regression examining the relationship between RNT, depression and anxiety outcomes overall and within subgroups of intervention types

Relationship with RNT	Slope coefficient (95% CI)	*Z* value	*p* value
All interventions			
Depression outcomes	0.96 (0.02–1.90)	2.01	0.04
Anxiety outcomes	1.07 (−0.08 to 2.22)	1.83	0.07
RNT focused interventions^a^			
Depression outcomes	1.03 (0.16–1.90)	2.32	0.02
Anxiety outcomes	0.35 (0.12–2.59)	2.15	0.03
Non-RNT focused interventions^a^			
Depression outcomes	0.81 (−0.45 to 2.08)	1.26	0.21
Anxiety outcomes	−0.11 (−6.52 to 6.31)	−0.03	0.97

a Results reported are those with the outlier trial removed. See online Supplementary for results including this trial.

### Study quality

**Fig. 3.**
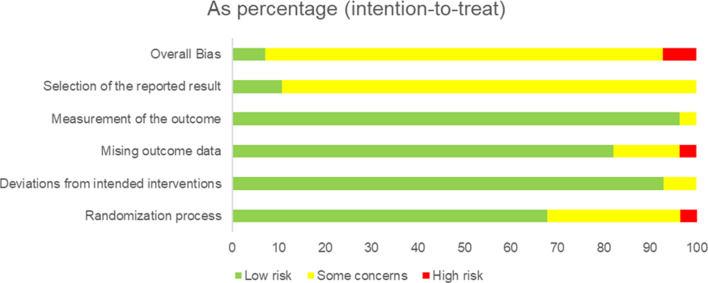
Cochrane risk of bias results.

The quality of included trials varied (see Fig. 3). Nineteen studies reported adequate sequence generation, two studies reported adequate randomisation procedures, 26 studies did not report problems with intervention deviations from protocol, 23 studies reported no issues with missing outcome data handling, and 27 studies reported no problems with outcome measurement. However, only two studies reported adequate selection of results reporting, with the primary risk of the remaining 27 studies being a lack of transparent pre-registration of analyses.

## Discussion

The aim of this review was to examine the effect of psychological treatment on RNT, depression and anxiety outcomes in young people. Significant effects favouring the intervention were found in a similar small to moderate range across all three outcomes. We also aimed to examine the relationship between RNT and depression and anxiety outcomes using meta-regression. Findings revealed a significant relationship between RNT and depression outcomes only across both intervention types, and with both depression and anxiety for RNT focused interventions only. Subgroup analyses revealed that interventions which targeted RNT specifically and those which did not had similar effects across all outcomes. However, interventions that primarily targeted the process of RNT through third wave approaches had greater effects in reducing RNT (which were in the large range) compared to those that targeted modifying the content of negative thoughts using second wave approaches (which were in the small range). Effect sizes also differed based on intervention delivery, with group-based interventions having overall very large effects, followed by moderate effects for individual, and small effects for digital and self-guided interventions.

The findings of this review are consistent with two prior meta-analyses of psychological interventions for depression and anxiety in adult populations. Spinhoven *et al*. (2018a, 2018b) examined the effect of cognitive behavioural therapy (CBT) approaches for depression on RNT, finding small to moderate effect sizes for both depression and RNT outcomes, which did not differ between interventions which targeted RNT specifically and those which did not. Similarly, Monteregge *et al*. (2020) examined the effect of interventions for anxiety in adults on RNT, finding moderate effects on RNT and anxiety, which were also comparable between RNT and non-RNT focused interventions. The current results indicate that these findings are consistent in youth populations with depression and anxiety, with small to moderate effects on depression, anxiety and RNT for both RNT and non-RNT focused interventions. Also consistent with these two prior reviews, we found a significant, positive relationship between RNT, depression and anxiety outcomes. In line with the findings of Spinhoven *et al*. (2018a, 2018b), this relationship was significant for interventions which targeted RNT specifically and not significant for general approaches. This finding supports the assertion that reducing RNT may be a pathway to improving depression and anxiety, particularly within RNT focused interventions. This finding is significant in the context of early intervention for youth mental health as targeting reduction in RNT as a putative mechanism driving adverse mental health outcomes in youth may offer a means of preventing illness progression (McGorry & Nelson, 2016; Shah *et al*., 2020).

The finding that RNT and non-RNT focused interventions had similar effects implies that reductions in RNT can be achieved using both targeted techniques and non-specific, general strategies (Monteregge *et al*., 2020). As highlighted by Monteregge *et al*., similar techniques are often used across different intervention types, and therefore approaches that do not target RNT directly may do so indirectly, and that this may occur via different pathways that lead to the same effect (e.g. Deacon *et al*., 2011). For example, traditional cognitive behavioural therapy such as in Richards *et al*. (2016) does not target RNT directly, however there is an explicit focus on modifying negative thoughts which may be repetitively focused on. Or in the case of self-monitoring in Kauer *et al*. (2012), noting when negative thoughts happen throughout the day may build awareness of tendencies to repeatedly focus on them, leading to decisions to refocus attention. This presents challenges for the categorisation of interventions into RNT and non-RNT focused subgroups, whereby different approaches could arguably fall into multiple categories. This is an inherent limitation to the current review, and presents challenges in interpreting results. This highlights one of the practical limitations of comparing psychological interventions in that they tend to include multiple components, making the isolation of active ingredients and pathways difficult (Mulder, Murray, & Rucklidge, 2017). Future research may benefit from employing more targeted methods, including: (1) operationalising intervention components and examining their mechanisms of action in isolation (Stein & Witkiewitz, 2020); (2) the inclusion of both outcome and processes measures, with mediation analyses to examine mechanisms (Kazdin, 2007); (3) examining changes in mechanisms and their relation with symptom outcomes in real time; and (4) the use of advanced trial designs such as micro-randomised controlled trials and dismantling studies which can compare and contrast different active ingredients (and their combinations) to isolate their effects (Collins *et al*., 2011; Stein & Witkiewitz, 2020).

Individual patient factors may also account for a lack of difference between intervention types, as well as a need more effective and targeted techniques for reducing RNT. The optimum approach for reducing RNT may differ depending on the person or context (Norcross & Wampold, 2011), and multiple transdiagnostic mechanisms may be functioning and interacting simultaneously. For example, younger adolescents with less developed meta cognitive abilities may be more suited to basic CBT techniques which provide education on thinking patterns prior to commencing process based techniques to change these patterns. This highlights the need for formulation-driven approaches to transdiagnostic interventions which consider the degree to which particular mechanism/s may be involved in maintaining symptoms and what approach may be best suited to the individual. In line with the transdiagnostic approach to treatment (Bullis *et al*., 2019), this means identifying individuals with elevated levels of RNT and delivering interventions directly targeting this problem, rather than on the basis of symptom severity or diagnosis. This level of personalisation may be best suited to idiographic methods such as ecological momentary assessment and intervention (Schueller, Aguilera, & Mohr, 2017; Trull & Ebner-Priemer, 2009), which involve the assessment and intervention of processes in real time, real world contexts. Novel intervention approaches which make use of these technologies to inform personalisation and tailoring based on time and context may be enable interventions to target disruption of transdiagnostic processes as they unfold in real time (Reininghaus, Depp, & Myin-Germeys, 2016).

Whilst the association between RNT, depression and anxiety outcomes provides further support for the significance of RNT in youth depression and anxiety, further research is needed to determine the nature of this relationship. To provide evidence of causality, mediation analysis is needed across three or more time points to show that changes in RNT precede improvements in outcomes (Kazdin, 2007). Whilst further research is clearly needed, individual trial results suggest that reducing RNT may be one of a number of active ingredients within psychological treatment of depression and anxiety (Norr, Allan, Macatee, Keough, & Schmidt, 2014). The interaction between these mechanisms should be a focus of future research. Results from individual trials also suggest that the significance of timing in intervention delivery (Modini & Abbott, 2017; Modini & Abbott, 2018). Processes such as RNT and mood states are known to be dynamic, fluctuating over time and in relation to surrounding contexts (Kircanski, Thompson, Sorenson, Sherdell, & Gotlib, 2015; Shiffman, 1999; Walz, Nauta, & aan het Rot, 2014). It is therefore understandable that the effectiveness of different intervention strategies may also change over time depending on specific contexts. Ecological momentary assessment methods in combination with ecological momentary intervention may be beneficial to examine when processes such as RNT are likely to be activated, the consequences this has on mood, and how this dynamic is impacted by different intervention strategies in the moment (Reininghaus *et al*., 2016; Trull & Ebner-Priemer, 2009).

A novel finding of this review is the observation that interventions which primarily target the process of RNT had larger effect sizes than those which target the content of the negative thoughts. This finding would suggest that RNT may benefit specifically from process-based approaches, however it is also important to recognise the aforementioned limitation regarding the challenges in categorising intervention types due to the inclusion of multiple components and pathways of action, resulting in poorly differentiated and heterogenous subgroups. For example, ABM attempts to modify focus on negative thoughts through attention training, therefore employing both process focused techniques with an inherent distinction between content (positive and negative thoughts). Sensitivity analyses was conducted to re-code AMB trials to a different subgroup, which did not affect the results favouring process focused interventions, providing some evidence of robustness. However, clearly further theoretical and empirical investigation using stronger methodologies to dismantle the pathways through which these interventions operate is needed.

The larger effect size for process interventions would suggest that third wave approaches that modify *how* a young person thinks may be more favourable than those which focused primarily on *what* they are thinking about. Further, this finding provides support for the broader construct of RNT as a process of repetitive thinking that captures the content specific forms of worry and rumination (Ehring & Watkins, 2008; Spinhoven *et al*., 2015). Arising from third wave approaches, most significantly ACT (Hayes, 2004), this finding aligns with theoretical arguments that emotional problems arise through problematic relations with thoughts and that therapeutic change requires awareness and adaptations to these reactions, rather than attempting to change the thought itself. Experimental studies comparing these techniques directly have typically found that both have a positive impact on negative thoughts in different ways. For example, cognitive defusion, a core technique within ACT, has been shown to decrease the believability and discomfort of negative thoughts whilst cognitive restructuring has been shown to have a greater effect on the perceived accuracy of negative thoughts (Deacon *et al*., 2011; Larsson, Hooper, Osborne, Bennett, & McHugh, 2016; Masuda *et al*., 2010). Future research would benefit from investigating the current finding by directly comparing different intervention types.

This review has several important limitations. Firstly, a relatively small number of heterogeneous trials were included, limiting interpretation of pooled effect sizes. Secondly, as previously mentioned, multicomponent interventions with likely numerous active ingredients made the isolation of effects challenging. This likely explains the high level of heterogeneity within subgroups. Further, as there were 17 unique interventions identified, comparing individual intervention types (e.g. mindfulness *v.* CBT approaches) was not possible. Further, the potential for interaction effects between subgroups cannot be ruled out. For example, different intervention types may have differential effects for those with heightened symptoms *v.* established clinical conditions, which may also vary based on delivery medium (e.g. self-guided app *v.* multi-session treatment with a psychologist). This is particularly apparent in this review where a broad range of interventions (including those for prevention and treatment) and clinical groups were included. Thirdly, meta-regression confirmed the association between RNT, depression and anxiety outcomes across trials, however formal mediation analyses was not possible to inform whether RNT is a pathway through which symptom improvement may occur. Fourth, studies showed a high level of bias in a lack of pre-registered analyses, however study quality was overall good. Studies of varying designs (i.e. clinical trials, experimental studies and pilot trials) were also combined, further complicating interpretation of overall effect size Fifth, the analysis was limited to post intervention effects and long-term outcomes were not evaluated. Finally, this review was conducted up until June 2020 and the possibility to further studies being published since this time cannot be discounted.

In conclusion, the current findings suggest that reducing RNT using a variety of psychological treatment approaches may help improve depression and anxiety in youth. The significant relationship between reductions in RNT and improvements in depression and anxiety highlights the clinical significance of RNT as a transdiagnostic target in psychological treatment for youth, although further research is needed to determine whether this relationship is causal. Findings suggest that process focused interventions maybe particularly effective for reducing RNT, providing support for third wave approaches and the theoretical relevance of RNT as an overarching process capturing both worry and rumination. However, further investigation is needed and an individualised approach to treatment planning is recommended (Weisz *et al*., 2015). Further research is needed to examine causal pathways and different treatment components using advanced methods to understand mechanisms of action. This may lead to more effective and personalised interventions which target processes such as RNT in real time.

## Supplementary material

For supplementary material accompanying this paper visit https://doi.org/10.1017/S0033291722003373. click here to view supplementary material
