# Supplementary material for: The effect of psychological treatment on repetitive negative thinking in youth depression and anxiety: a meta-analysis and meta-regression
Source: Psychol Med. 2022 Nov 14;53(1):6–16. doi: 10.1017/S0033291722003373 (PMC9875014; doi:10.1017/S0033291722003373)
Supplement: Supplementary file 1 [file S0033291722003373sup001.docx]

**Supplementary material**

**Supplementary 1.** PRISMA checklist

| **Section/topic** | **#** | **Checklist item** | **Reported on page #** |
| --- | --- | --- | --- |
| **TITLE** | | |  |
| Title | 1 | Identify the report as a systematic review, meta-analysis, or both. | 1 |
| **ABSTRACT** | | |  |
| Structured summary | 2 | Provide a structured summary including, as applicable: background; objectives; data sources; study eligibility criteria, participants, and interventions; study appraisal and synthesis methods; results; limitations; conclusions and implications of key findings; systematic review registration number. | 2 |
| **INTRODUCTION** | | |  |
| Rationale | 3 | Describe the rationale for the review in the context of what is already known. | 3-6 |
| Objectives | 4 | Provide an explicit statement of questions being addressed with reference to participants, interventions, comparisons, outcomes, and study design (PICOS). | 7 |
| **METHODS** | | |  |
| Protocol and registration | 5 | Indicate if a review protocol exists, if and where it can be accessed (e.g., Web address), and, if available, provide registration information including registration number. | 7 |
| Eligibility criteria | 6 | Specify study characteristics (e.g., PICOS, length of follow-up) and report characteristics (e.g., years considered, language, publication status) used as criteria for eligibility, giving rationale. | 7-8 |
| Information sources | 7 | Describe all information sources (e.g., databases with dates of coverage, contact with study authors to identify additional studies) in the search and date last searched. | 7 |
| Search | 8 | Present full electronic search strategy for at least one database, including any limits used, such that it could be repeated. | 7, supplementary |
| Study selection | 9 | State the process for selecting studies (i.e., screening, eligibility, included in systematic review, and, if applicable, included in the meta-analysis). | 7 |
| Data collection process | 10 | Describe method of data extraction from reports (e.g., piloted forms, independently, in duplicate) and any processes for obtaining and confirming data from investigators. | 7-8 |
| Data items | 11 | List and define all variables for which data were sought (e.g., PICOS, funding sources) and any assumptions and simplifications made. | 7-8 |
| Risk of bias in individual studies | 12 | Describe methods used for assessing risk of bias of individual studies (including specification of whether this was done at the study or outcome level), and how this information is to be used in any data synthesis. | 9 |
| Summary measures | 13 | State the principal summary measures (e.g., risk ratio, difference in means). | 8 |
| Synthesis of results | 14 | Describe the methods of handling data and combining results of studies, if done, including measures of consistency (e.g., I^2^) for each meta-analysis. | 8 |
| Risk of bias across studies | 15 | Specify any assessment of risk of bias that may affect the cumulative evidence (e.g., publication bias, selective reporting within studies). | 9 |
| Additional analyses | 16 | Describe methods of additional analyses (e.g., sensitivity or subgroup analyses, meta-regression), if done, indicating which were pre-specified. | 8 |
| **RESULTS** |  |  |  |
| Study selection | 17 | Give numbers of studies screened, assessed for eligibility, and included in the review, with reasons for exclusions at each stage, ideally with a flow diagram. | 9-10 |
| Study characteristics | 18 | For each study, present characteristics for which data were extracted (e.g., study size, PICOS, follow-up period) and provide the citations. | 9, supplementary |
| Risk of bias within studies | 19 | Present data on risk of bias of each study and, if available, any outcome level assessment (see item 12). | 10 |
| Results of individual studies | 20 | For all outcomes considered (benefits or harms), present, for each study: (a) simple summary data for each intervention group (b) effect estimates and confidence intervals, ideally with a forest plot. | 10-11, Supplementary |
| Synthesis of results | 21 | Present results of each meta-analysis done, including confidence intervals and measures of consistency. | 10 |
| Risk of bias across studies | 22 | Present results of any assessment of risk of bias across studies (see Item 15). | 11 |
| Additional analysis | 23 | Give results of additional analyses, if done (e.g., sensitivity or subgroup analyses, meta-regression [see Item 16]). | 10-11 |
| **DISCUSSION** |  |  |  |
| Summary of evidence | 24 | Summarize the main findings including the strength of evidence for each main outcome; consider their relevance to key groups (e.g., healthcare providers, users, and policy makers). | 12-15 |
| Limitations | 25 | Discuss limitations at study and outcome level (e.g., risk of bias), and at review-level (e.g., incomplete retrieval of identified research, reporting bias). | 15-16 |
| Conclusions | 26 | Provide a general interpretation of the results in the context of other evidence, and implications for future research. | 16 |
| **FUNDING** |  |  |  |
| Funding | 27 | Describe sources of funding for the systematic review and other support (e.g., supply of data); role of funders for the systematic review. | 16 |

**Supplementary 2.** Additional information on inclusion criteria and search details of Orygen Evidence Finder

The aim of the search strategy for the Orygen Evidence Finder was to identify all trials, and reviews of trials, investigating any intervention strategy aiming to prevent or treat a number of mental health conditions (or emerging conditions) in young people, compared to a control/comparison condition. The search terms were constructed for each condition (i.e. anxiety disorders, psychotic disorders etc) for each database, and standard inclusion criteria were adopted across all studies, except for mental health condition which was unique. The current review identified all trials within the depression and anxiety categories, and adopted unique inclusion/exclusion criteria within the studies identified. These unique inclusion/exclusion are detailed in the main paper. Below are further details of the broader search strategy and inclusion/exclusion from the Orygen Evidence Finder. For brevity, an example from the Ovid Medline database is provided and details of each database can be provided on request, or readers can refer to the primary paper detailing the method adopted by the Orygen Evidence Finder:

De Silva, S., Bailey, A. P., Parker, A. G., Montague, A. E., & Hetrick, S. E. (2018). Open‐access evidence database of controlled trials and systematic reviews in youth mental health. *Early Intervention in Psychiatry, 12*(3), 474-477.

**Inclusion criteria depression conditions:**

*1. Depression definition used throughout*

1. **Depression diagnosis** established according to DSM/ICD or other classification system e.g., Major Depressive Disorder, dysthymia etc.
2. **Depression symptoms** measured on a self-report or observer-rated depression symptom/severity scale.
3. **Depression risk factors** e.g., familial risk, history of significant life event etc.

*2.a Participants: Age*

**Include** trials of children, adolescents, young people (or related descriptor), where:

1. mean age of at least one group is ≥ 6 **AND** < 26 years.

**IF** mean age is not reported, use:

1. median OR range (min & max is within above age range), **OR**
2. where authors use a youth-related sample or population descriptor.

**Exclude:**

1. mean age < 6 OR ≥ 26 years.
2. age can’t be determined (i.e. age AND youth-related descriptor, not reported)

*2.b Participants: Depression status at recruitment*

**Universal Prevention, include** trials recruiting:

1. healthy young people with no known depression risk factors, **OR**
2. entire populations regardless of depression risk (e.g., schools, communities)

**At-Risk Prevention/Treatment, include** trials recruiting:

1. young people with any known risk factor for depression*
2. young people with any level of depression symptoms, excluding established diagnosis

**Disorder Established Treatment, include** trials recruiting:

1. young people with an established depression diagnosis

**Treatment Resistant, include** trials recruiting:

1. young people with at least 1 previously unsuccessful depression intervention*

**Relapse Prevention, include** trials recruiting:

1. young people who have recovered or responded to a previous depression intervention*

**Comorbid/co-occurring conditions, include** trials recruiting:

1. young people with depression risk, symptoms or diagnosis **AND** a comorbid/co-occurring mental health disorder, symptom or risk factor.
2. Comorbid recruitment must be from a mapping disorder category (depression, anxiety, bipolar, eating disorders, substance use, psychosis)

**Exclude** trials explicitly recruiting young people on the basis of any:

1. primary physiological/medical condition (e.g., hyperthyroidism, cardio-vascular disease etc.), neurodevelopmental disorder (e.g., intellectual disability, ADHD, autism etc.), or pregnancy (e.g., pre/post/peri-natal).
2. Note: Other non-recruitment comorbidities that may be present at baseline are not exclusionary, so long as they are NOT the basis for recruitment.

* = author definition accepted

*3. Study design*

**Intervention trials.**

**Include:**

1. randomised controlled/comparative trial (RCT), **OR**
2. non-randomised controlled/comparative trial (aka controlled clinical trial, CCT),
3. including parallel, cluster and cross-over RCT/CCT designs.
4. Participants must be purposefully assigned to at least two conditions, **AND** be drawn from the same target population/group.

**Exclude** the following designs**:**

1. cohort, case-control, single-group pre-post, historical control & chart review, **OR**
2. where youth from different pops. are compared (depression vs. healthy control)

**Systematic reviews of intervention trials.**

**Include** reviews that:

1. include intervention trials meeting all mapping inclusion criteria, **AND**
2. report a search strategy (incl. search terms **AND** search databases), OR where the review is described as a "systematic review" or "meta-analysis”.
3. **IF** both adult **AND** youth trials are included, a synthesis of youth trials must be reported (e.g., narrative subsection, meta-analysis subgroup).
4. **IF** both RCT/CCTs **AND** other designs (e.g., uncontrolled studies) are included, a synthesis of RCT/CCTs must be reported.

*4. Intervention*

**Include** trials of any psychological, biological, complementary & alternative, service delivery & improvement **OR** other intervention strategy:

1. broadly aimed at reducing risk for, preventing, or treating depression*, AND
2. delivered to young people, OR delivered to people/services/communities involved in supporting young people

**Exclude**:

1. non-intervention studies

*5. Comparison*

**Include** trials comparing an intervention to any:

1. control condition (wait-list, no-treatment, placebo etc.), without limit, **AND/OR**
2. comparison condition, including interventions, without limit.

**Exclude** trials:

1. without a control/comparison condition

*6. Outcomes*

**Include** trials reporting:

1. any depression outcomes (e.g., diagnosis, symptom/severity scales etc.), AND
2. young person-specific (outcomes reported by them, or by others, about them)

**Exclude** studies where:

1. no depression outcomes are identified/reported (e.g., protocols, cost-effectiveness analyses or conference abstracts with no outcomes reported),
2. no young person outcomes are reported (e.g., parent/clinician attitudes)

*7. Other*

**Include:**

1. conference proceedings, letters to the editor and other publications reporting trials or reviews, so long as sufficient information is reported to fulfill all inclusion criteria, notably reporting of outcome data.
2. secondary publications reporting new outcomes not previously reported in the primary publication (e.g., 6-month follow-up data, other depression outcomes not previously reported)

**Exclude:**

1. non-English language publications
2. dissertations, book chapters and other non-peer reviewed reports
3. secondary pubs/duplicate records where no new outcome data is reported

**Inclusion criteria anxiety conditions:**

*1. Anxiety definition used throughout*

1. **Anxiety diagnosis** established according to DSM/ICD or other classification system. Included disorder categories are based on DSM-IV-TR (current at time evidence map was created): Social Anxiety Disorder, Generalised Anxiety Disorder, Specific Phobia, Panic Disorder, Acute Stress Disorder, Obsessive Compulsive Disorder, Post-Traumatic Stress Disorder.
2. **Anxiety symptoms** measured on a self-report or observer-rated anxiety symptom/severity scale.
3. **Anxiety risk factors** e.g., familial risk, history of significant life event etc.

*2.a Participants: Age*

**Include** trials of children, adolescents, young people (or related descriptor), where:

1. mean age of at least one group is ≥ 6 **AND** < 26 years.

**IF** mean age is not reported, use:

1. median OR range (min & max is within above age range), **OR**
2. where authors use a youth-related sample or population descriptor.

**Exclude:**

1. mean age < 6 OR ≥ 26 years.
2. age can’t be determined (i.e. age AND youth-related descriptor, not reported)

*2.b Participants: Anxiety status at recruitment*

**Universal Prevention, include** trials recruiting:

1. healthy young people with no known anxiety risk factors, **OR**
2. entire populations regardless of anxiety risk (e.g., schools, communities)

**At-Risk Prevention/Treatment, include** trials recruiting:

1. young people with any known risk factor for anxiety*
2. young people with any level of anxiety symptoms, excluding established diagnosis

**Disorder Established Treatment, include** trials recruiting:

1. young people with an established anxiety diagnosis

**Treatment Resistant, include** trials recruiting:

1. young people with at least 1 previously unsuccessful anxiety intervention*

**Relapse Prevention, include** trials recruiting:

1. young people who have recovered or responded to a previous anxiety intervention*

**Comorbid/co-occurring conditions, include** trials recruiting:

1. young people with anxiety risk, symptoms or diagnosis **AND** a comorbid/co-occurring mental health disorder, symptom or risk factor.
2. Comorbid recruitment must be from a mapping disorder category (depression, suicide/self-harm, bipolar, eating disorders, substance use, psychosis)

**Exclude** trials explicitly recruiting young people on the basis of any:

1. primary physiological/medical condition (e.g., hyperthyroidism, cardio-vascular disease etc.), neurodevelopmental disorder (e.g., intellectual disability, ADHD, autism etc.), or pregnancy (e.g., pre/post/peri-natal).
2. Note: Other non-recruitment comorbidities that may be present at baseline are not exclusionary, so long as they are NOT the basis for recruitment.

* = author definition accepted

*3. Study design*

**Intervention trials.**

**Include:**

1. randomised controlled/comparative trial (RCT), **OR**
2. non-randomised controlled/comparative trial (aka controlled clinical trial, CCT),
3. including parallel, cluster and cross-over RCT/CCT designs.
4. Participants must be purposefully assigned to at least two conditions, **AND** be drawn from the same target population/group.

**Exclude** the following designs**:**

1. cohort, case-control, single-group pre-post, historical control & chart review, **OR**
2. where youth from different pops. are compared (anxiety vs. healthy control)

**Systematic reviews of intervention trials.**

**Include** reviews that:

1. include intervention trials meeting all mapping inclusion criteria, **AND**
2. report a search strategy (incl. search terms **AND** search databases), OR where the review is described as a "systematic review" or "meta-analysis”.
3. **IF** both adult **AND** youth trials are included, a synthesis of youth trials must be reported (e.g., narrative subsection, meta-analysis subgroup).
4. **IF** both RCT/CCTs **AND** other designs (e.g., uncontrolled studies) are included, a synthesis of RCT/CCTs must be reported.

*4. Intervention*

**Include** trials of any psychological, biological, complementary & alternative, service delivery & improvement **OR** other intervention strategy:

1. broadly aimed at reducing risk for, preventing, or treating anxiety*, AND
2. delivered to young people, OR delivered to people/services/communities involved in supporting young people

**Exclude**:

1. non-intervention studies

*5. Comparison*

**Include** trials comparing an intervention to any:

1. control condition (wait-list, no-treatment, placebo etc.), without limit, **AND/OR**
2. comparison condition, including interventions, without limit.

**Exclude** trials:

1. without a control/comparison condition

*6. Outcomes*

**Include** trials reporting:

1. any anxiety outcomes (e.g., diagnosis, symptom/severity scales etc.), AND
2. young person-specific (outcomes reported by them, or by others, about them)

**Exclude** studies where:

1. no anxiety outcomes are identified/reported (e.g., protocols, cost-effectiveness analyses or conference abstracts with no outcomes reported),
2. no young person outcomes are reported (e.g., parent/clinician attitudes)

*7. Other*

**Include:**

1. conference proceedings, letters to the editor and other publications reporting trials or reviews, so long as sufficient information is reported to fulfill all inclusion criteria, notably reporting of outcome data.
2. secondary publications reporting new outcomes not previously reported in the primary publication (e.g., 6-month follow-up data, other anxiety outcomes not previously reported)

**Exclude:**

1. non-English language publications
2. dissertations, book chapters and other non-peer reviewed reports
3. secondary pubs/duplicate records where no new outcome data is reported

**Search syntax for Medline depressive conditions**

| **#** | **Query** |
| --- | --- |
| 1 | *Depression/ or *Depressive Disorder/ or *Depressive Disorder, Major/ or *Dysthymic Disorder/ or *Mood Disorders/ |
| 2 | clinical trial.pt. |
| 3 | clinical trial*.ti. or clinical trial*.ab. or clinical trial/ or clinical trial*.rn. |
| 4 | random*.ti. or random*.ab. or random*/ or random*.rn. |
| 5 | placebo*.ti. or placebo*.ab. |
| 6 | groups.ti. or groups.ab. |
| 7 | meta-analysis.pt. |
| 8 | meta-analysis.ti. or meta-analysis.ab. or meta-analysis/ or meta-analysis.rn. |
| 9 | (meta-anal* or metaanal* or meta analy*).ti. or (meta-anal* or metaanal* or meta analy*).ab. |
| 10 | review.pt. |
| 11 | systematic review.ti. or systematic review.ab. |
| 12 | guideline.pt. |
| 13 | exp Guideline/ |
| 14 | Health Planning Guidelines/ |
| 15 | guideline*.ti. or guideline*.ab. |
| 16 | consensus.ti. or consensus.ab. |
| 17 | or/2-16 |
| 18 | 1 and 17 |
| 19 | Adolescent/ |
| 20 | Adult/ |
| 21 | 19 or 20 |
| 22 | 18 and 21 |
| 23 | Adolescent/ or Adult/ or exp Infant/ or exp Child/ or exp Aged/ or Middle Aged/ |
| 24 | 18 not 23 |
| 25 | 22 or 24 |
| 26 | limit 25 to english |
| 27 | limit 26 to yr="" |

**Search syntax for Medline anxiety conditions**

| **#** | **Query** |
| --- | --- |
| 1 | Anxiety/ or exp Anxiety Disorders/ |
| 2 | clinical trial.pt. |
| 3 | clinical trial*.ti. or clinical trial*.ab. or clinical trial/ or clinical trial*.rn. |
| 4 | random*.ti. or random*.ab. or random*.rn. |
| 5 | placebo*.ti. or placebo*.ab. |
| 6 | or/2-5 |
| 7 | meta-analysis.pt. |
| 8 | meta-analysis.ti. or meta-analysis.ab. or meta-analysis/ or meta-analysis.rn. |
| 9 | (meta-anal* or metaanal* or meta analy*).ti. or (meta-anal* or metaanal* or meta analy*).ab. |
| 10 | review.pt. |
| 11 | systematic review.ti. or systematic review.ab. |
| 12 | or/7-11 |
| 13 | guideline.pt. |
| 14 | exp Guideline/ |
| 15 | Health Planning Guidelines/ |
| 16 | guideline*.ti. or guideline*.ab. |
| 17 | consensus.ti. or consensus.ab. |
| 18 | or/13-17 |
| 19 | 6 or 12 or 18 |
| 20 | 1 and 19 |
| 21 | exp child/ |
| 22 | Adolescent/ |
| 23 | Adult/ |
| 24 | or/21-23 |
| 25 | 20 and 24 |
| 26 | Adolescent/ or Adult/ or exp Infant/ or exp Child/ or exp Aged/ or Middle Aged/ |
| 27 | 20 not 26 |
| 28 | 25 or 27 |
| 29 | exp Animals/ |
| 30 | exp Humans/ |
| 31 | 29 not 30 |
| 32 | 28 not 31 |
| 33 | limit 32 to english |
| 34 | limit 33 to yr="" |

Relevant subject headings for each database were used where appropriate. Additional searching included all reference lists of included systematic reviews and meta-analyses that were hand-searched for studies relevant to the map.

**Supplementary 3.** Characteristics of included studies

| **Authors and country** | **Population** | | | | **Study design** | | | | **Intervention** | | |
| --- | --- | --- | --- | --- | --- | --- | --- | --- | --- | --- | --- |
|  | **Sample size** | **Demographics** | **Clinical condition** | **Recruitment context** | **Type** | **Comparisons** | **Timepoints** | **Outcome measure** | **Type and delivery** | **RNT or non-RNT focused** | **Process or content focused** |
| Bernal-Manrique et al (2020)  *Columbia* | 42 | *Age:* 14.52(1.67)  *%Female:* 71% | Elevated depression and anxiety | School | Clinical trial | Waitlist control | Pre, post | *RNT:* PTQ-C  *Depression:* DASS – 21  *Anxiety:* NA | *Mode:* Group  *Length:* 3 sessions  *Treatment:* RNT-focused ACT | RNT-focused | Process |
| deVoogd et al (2017a)  *The Netherlands* | 150 | *Age:*  15.68(1.33)  *%Female:* 63% | Elevated depression and anxiety | School | Experimental study | Active control;  Neutral control | Pre, 4-week post, 3-month FU, 6-month FU | *Depression:* CDI  *Anxiety:* SCARED  *RNT:* PTQ | *Mode:* Computerised self-guided  *Length:* 8 sessions  *Treatment:* ABM | RNT-focused | Content |
| deVoogd et al (2017b)  *United Kingdom* | 121 | *Age:* 14.45(1.53)  *%Female:* 66.7% | Elevated depression and anxiety | School | Experimental study | Active control;  Neutral control | Pre, 4-week post, 3-month FU, 6-month FU | *Depression:* CDI  *Anxiety:* SCARED  *RNT:* PTQ | *Mode:* Computerised self-guided  *Length:* 8 sessions  *Treatment:* AMB | RNT-focused | Content |
| Grol et al (2018)  *United Kingdom* | 81 | *Age:* 23.17(4.01)  *%Female:* 86.7% | Elevated depression and anxiety | University | Experimental study | Active control;  Sham training | Pre, 11-day post | *Anxiety:* STAI-T  *RNT:* PSWQ | *Mode:* Computerised self-guided  *Length:* 10 sessions  *Treatment:* ABM | RNT-focused | Content |
| Idsoe et al (2019)  *Norway* | 228 | *Age:* 16·70(1·14)  *%Female:* 88% | Elevated depression and anxiety | Community | Clinical trial | Treatment as usual | Screening, Pre-test, Post-test | *Anxiety:* CES-D  *RNT:* RRS | *Mode:* Group  *Length:* 8 sessions  *Treatment:* CBT | Non RNT-focused | Content |
| Kauer et al (2012)  *Australia* | 118 | *Age:* 17.95(3.20)  *%Female:* 73% | Clinical depression | Community | Clinical trial | Active control | Pre, post, 6-week FU | *Depression:* DASS-D  *RNT:* RRS | *Mode:* Mobile  *Length:* Continuous access (between 2-4 weeks)  *Treatment:* Self-monitoring | Non RNT-focused | Content |
| Kocovski et al (2019)  *Canada* | 152 | *Age:* 23.95(6.72)  *%Female:* 73.68% | Elevated depression and anxiety | Community | Clinical trial | Waitlist control | Pre, post, 6-week FU | *Depression:* BDI-II  *Anxiety:* LSAS, SPIN, SA-AAQ-SF  *RNT*: PEPI, CFQ | *Mode:* Self-help book  *Length:* Continuous access  *Treatment:* ACT | RNT-focused | Process |
| LaFreniere and Newman (2016)  *USA* | 51 | *Age:* 18.86 (1.07)  *%Female:* 84% | Clinical anxiety | University | Clinical trial | Active control | Pre, 10-day post, 20-day FU | *Anxiety:* GAD Questionnaire  for DSM-IV  *RNT:* PSWQ, MCQ | *Mode:* Mobile  *Length:* Continuous access (10 days)  *Treatment:* Worry monitoring | RNT-focused | Content |
| Lytle et al (2002)  *USA* | 48 | *Age:* 18.89(1.64)  *%Female:* 75% | Elevated depression and anxiety | University | Experimental study | Active control | Pre, post | *Depression:* BDI-II  *Anxiety:* STAI-T  *RNT*: PSWQ | *Mode:* Clinician delivered  *Length:* 1 session  *Treatment:* EMDR | RNT-focused | Content |
| McDermott & Cougle (2021) | 50 | *Age:* 19.02(1.64)  *%Female: 92*% | Elevated anxiety | University | Experimental study | Waitlist | Pre, post | *Depression:* BDI-II  *Anxiety:* DASS-A  *RNT:* PSQW, DERS Worry, WDQ | *Mode:*  Computerised self-guided  *Length: 6* sessions  *Treatment:* Worry disengagement training | RNT-focused | Process |
| McEvoy et al (2017)  *Australia* | 81 | *Age:* 23.60(7.66)  *%Female:* 80.2% | Elevated depression and anxiety | University | Experimental study | Active control | Pre, post | *Anxiety:* STICSA  *RNT*: UTS | *Mode:* Computerised self-guided  *Length:* 1 session  *Treatment:* Attention training; Mindfulness | RNT-focused | Process |
| McIndoo et al (2016)  *USA* | 50 | *Age:* 19.20(1.67)  *%Female:* 62% | Elevated depression and anxiety | University | Clinical trial | Waitlist control | Pre, post, 1-month FU | *Depression:* BDI-II, HAM-D  *Anxiety:* BAI  *RNT*: RRS | *Mode:* Clinician delivered  *Length:* 4 sessions  *Treatment:*  BA;  Mindfulness | RNT-focused  Non RNT-focused | Process |
| McIntosh and Crino (2013)  *Australia* | 9 | *Age:* 22.56(6.64)  *%Female:* 80.2% | Elevated depression and anxiety | University | Pilot trial | Active control | Pre, post, 3-month follow-up | *Depression:* DASS  *RNT*: PSWQ | *Mode:* Clinician delivered  *Length:* 4 sessions  *Treatment:* CBT exposure therapy | RNT-focused | Content |
| Modini and Abbott (2018)  *Australia* | 53 | *Age:* 23.95(6.72)  *%Female:* 67.9% | Clinical anxiety | University | Pilot trial | Non-active control | Pre, day 1-4, post | *Anxiety:* SAR  *RNT:*  TQ | *Mode:* Computerised self-guided  *Length:* 1 session  *Treatment:* Mindfulness | RNT-focused | Process |
| Modini and Abbott (2017)  *Australia* | 49 | *Age:* 19.89(3.66)  *%Female:* 75% | Clinical anxiety | University | Pilot trial | Non-active control | Pre, post, 1-week FU | *Anxiety:* SAR  *RNT:*  AFQ; TQ | *Mode:* Clinician delivered  *Length:* 1 session  *Treatment:* CBT | RNT-focused | Content |
| Mogoase et al (2013)  *Romania* | 42 | *Age:* 22.87(4.27)  *%Female:* 95% | Elevated depression and anxiety | University | Pilot trial | Waitlist control | Pre, post | *Depression:* BDI-II  *RNT: RRS* | *Mode:* Computerised self-guided  *Length:* 7 sessions  *Treatment:* Concreteness training | RNT-focused | Process |
| Norr et al (2014)  *USA* | 104 | *Age:* 18.90(1.42)  *%Female:* 83.7% | Elevated depression and anxiety | University | Clinical trial | Active control | Pre, 1-week post, 1-month FU | *Depression:* BDI-II  *Anxiety:* ASI-3; BAI  *RNT: PSWQ* | *Mode:* Clinician delivered  *Length:* 1 session  *Treatment:* Anxiety sensitivity training | Non RNT-focused | Content |
| Richards et al (2014)  *Ireland* | 137 | *Age:* 23.82(7.05)  *%Female:* 77% | Elevated depression and anxiety | University | Clinical trial | Waitlist control | Pre, post, 3-month FU | *Depression:* BDI-II  *Anxiety:* GAD-7  *RNT: PSWQ* | *Mode:* Computerised self-guided  *Length:* Continuous access (6 weeks)  *Treatment:* CBT | Non RNT-focused | Content |
| Sass et al (2017)  *USA* | 41 | *Age:* 19.80(2.40)  *%Female:* 70.7% | Elevated depression and anxiety | University | Experimental study | Placebo control | Pre, post | *Depression:*  MASQ-AD  *Anxiety:* MASQ-AD  *RNT:* PSWQ | *Mode:* Computerised self-guided  *Length:* 1 session  *Treatment:* ABM | RNT-focused | Content |
| Short & Schmidt (2020) | 61 | *Age:*  19.43 (2.04)  *%Female:* 84% | Elevated anxiety | University | Experimental study | Active control | Pre, post, 1 month FU | *Anxiety:*  BAI  *RNT:*  PSQW | *Mode:*  Computerised self-guided  *Length:*  1 session  *Treatment:* CBT for Insomnia | Non-RNT focused | Content |
| Skodzik et al (2018)  *Germany* | 112 | *Age:* 22.02(3.38)  *%Female:* 84.8% | Elevated depression and anxiety | University | Experimental study | Waitlist control | Pre, post, 5-week FU | *Depression:* PHQ-9  *Anxiety:* STAI-T, BAI  *RNT:* PSWQ | *Mode:* Computerised self-guided  *Length:* Continuous access (7 days)  *Treatment:* Mental imagery training | RNT-focused | Process |
| Teng et al (2019)  *Taiwan* | 82 | *Age:* 21.47(NR)  *%Female:* 74% | Elevated depression and anxiety | Community | Experimental study | Waitlist control | Pre, during at weeks 2-4, FU | *Depression:* BDI-II  *Anxiety:* STAI-T, BAI  *RNT:* PSWQ | *Mode:* Mobile  *Length:* 12 sessions  *Treatment:* ABM | RNT-focused | Content |
| Topper et al (2017)  *The Netherlands* | 251 | *Age:* 17.45(2.09)  *%Female:* 83.63% | Elevated depression and anxiety | University | Clinical trial | Waitlist control | Pre, post, 3-month and 12-month FU | *Depression:* BDI-II; PHQ-9  *Anxiety:* MASQ-AD-30; GAD-Q-IV  *RNT:* PSWQ; RRS; PTQ | *Mode:* Online  *Length:* 6 sessions  *Treatment:* RFCBT | RNT-focused | Process |
| Vrijsen et al (2019)  The Netherlands | 201 | *Age:* 19(1.30)  *%Female:* 84% | Elevated depression and anxiety | University | Experimental study | Neutral control | Pre, post | *Depression:* BDI-II  *Anxiety:* BAI  *RNT:* RRS; MSRI | *Mode:* Computerised self-guided  *Length:* 2 sessions  *Treatment:* ABM | RNT-focused | Content |
| Wilkinson and Goodyer (2008)  *United Kingdom* | 61 | *Age:* 15.13(1.07)  *%Female:* 70.5% | Clinical depression | Outpatient clinic | Clinical trial | Treatment as usual | Pre, 12-week post, 30-week FU | *Depression:* MFQ  *RNT*: RDQ | *Mode:* Clinician delivered  *Length:* 28 sessions  *Treatment:* CBT;  CBT + SSRI | Non RNT-focused | Content |
| Wong et al (2020) | 136 | *Age:*  12-14: 28.1%  15-16: 44.4%  17-19: 33.3%  *%Female:* 56.6% | Elevated anxiety | School | Clinical trial | Active control | Pre, post, 6-month FU | *Anxiety:*  HADS-A  *RNT:* CERQ-Rumination | *Mode:* Clinician delivered  *Length:* 6 sessions  *Treatment:* CBT | Non RNT-focused | Content |
| Yang et al (2016)  *China* | 45 | *Age:* 15.09(1.51)  *%Female:* 45.4% | Elevated depression and anxiety | School | Clinical trial | Neutral control | Pre-training, post-neutral ABM, 7-week follow up, pre-positive ABM, post-positive ABM | Depression: HAM-D; CES-D  Anxiety: STAI-T  RNT: RRS | *Mode:* Computerised self-guided  *Length:* 8 sessions  *Treatment:* ABM | RNT-focused | Content |
| Zemenstani et al (2016)  *Iran* | 61 | *Age:* 24.42(NR)  *%Female:* 60% | Clinical depression | University | Clinical trial | Waitlist control | Pre, post, 3-month FU | *Depression: BDI-II*  *Anxiety:* BAI  *RNT:* CERQ | *Mode:* Group  *Length:* 8 sessions  *Treatment:* MCT; BA | RNT-focused | Process |

**Supplementary 4: References of included studies**

1. Bernal-Manrique, K. N., García-Martín, M. B., & Ruiz, F. J. (2020). Effect of acceptance and commitment therapy in improving interpersonal skills in adolescents: A randomized waitlist control trial. Journal of Contextual Behavioral Science, 17, 86-94.
2. De Voogd, E. L., De Hullu, E., Burnett Heyes, S., Blackwell, S. E., Wiers, R. W., & Salemink, E. (2017a). Imagine the bright side of life: A randomized controlled trial of two types of interpretation bias modification procedure targeting adolescent anxiety and depression. PLoS One, 12(7), e0181147.
3. De Voogd, E. L., Wiers, R. W., & Salemink, E. (2017b). Online visual search attentional bias modification for adolescents with heightened anxiety and depressive symptoms: A randomized controlled trial. Behaviour Research and Therapy, 92, 57-67.
4. Grol, M., Schwenzfeier, A. K., Stricker, J., Booth, C., Temple-McCune, A., Derakshan, N., ... & Fox, E. (2018). The worrying mind in control: An investigation of adaptive working memory training and cognitive bias modification in worry-prone individuals. Behaviour Research and Therapy, 103, 1-11.
5. Idsoe, T., Keles, S., Olseth, A. R., & Ogden, T. (2019). Cognitive behavioral treatment for depressed adolescents: results from a cluster randomized controlled trial of a group course. BMC Psychiatry, 19(1), 1-17.
6. Kauer, S. D., Reid, S. C., Crooke, A. H. D., Khor, A., Hearps, S. J. C., Jorm, A. F., ... & Patton, G. (2012). Self-monitoring using mobile phones in the early stages of adolescent depression: randomized controlled trial. Journal of Medical Internet Research, 14(3), e67.
7. Kocovski, N. L., Fleming, J. E., Blackie, R. A., MacKenzie, M. B., & Rose, A. L. (2019). Self-help for social anxiety: Randomized controlled trial comparing a mindfulness and acceptance-based approach with a control group. Behavior Therapy, 50(4), 696-709.
8. LaFreniere, L. S., & Newman, M. G. (2016). A brief ecological momentary intervention for generalized anxiety disorder: A randomized controlled trial of the Worry Outcome Journal. Depression and Anxiety, 33(9), 829-839.
9. Lytle, R. A., Hazlett-Stevens, H., & Borkovec, T. D. (2002). Efficacy of eye movement desensitization in the treatment of cognitive intrusions related to a past stressful event. Journal of Anxiety Disorders, 16(3), 273-288.
10. McDermott, K. A., & Cougle, J. R. (2021). Disengagement training for the treatment of pathological worry: A preliminary test. Behavior Therapy, 52(1), 86-98.
11. McEvoy, P. M., Graville, R., Hayes, S., Kane, R. T., & Foster, J. K. (2017). Mechanisms of change during attention training and mindfulness in high trait-anxious individuals: A randomized controlled study. Behavior Therapy, 48(5), 678-694.
12. McIndoo, C. C., File, A. A., Preddy, T., Clark, C. G., & Hopko, D. R. (2016). Mindfulness-based therapy and behavioral activation: a randomized controlled trial with depressed college students. Behaviour Research & Therapy, 77, 118-128.
13. McIntosh, C., & Crino, R. (2013). Towards a unified worry exposure protocol for generalised anxiety disorder: A pilot study. Behaviour Change, 30(3), 210-225.
14. Modini, M., & Abbott, M. J. (2018). Banning pre-event rumination in social anxiety: A preliminary randomized trial. Journal of Behavior Therapy and Experimental Psychiatry, 61, 72-79.
15. Modini, M., & Abbott, M. J. (2017). Negative rumination in social anxiety: A randomised trial investigating the effects of a brief intervention on cognitive processes before, during and after a social situation. Journal of Behavior Therapy and Experimental Psychiatry, 55, 73-80.
16. Mogoaşe, C., Brăilean, A., & David, D. (2013). Can concreteness training alone reduce depressive symptoms? A randomized pilot study using an internet-delivered protocol. Cognitive Therapy and Research, 37(4), 704-712.
17. Norr, A. M., Allan, N. P., Macatee, R. J., Keough, M. E., & Schmidt, N. B. (2014). The effects of an anxiety sensitivity intervention on anxiety, depression, and worry: Mediation through affect tolerances. Behaviour Research and Therapy, 59, 12-19.
18. Richards, D., Timulak, L., Rashleigh, C., McLoughlin, O., Colla, A., Joyce, C., ... & Anderson-Gibbons, M. (2016). Effectiveness of an internet-delivered intervention for generalized anxiety disorder in routine care: a randomised controlled trial in a student population. Internet Interventions, 6, 80-88.
19. Sass, S. M., Evans, T. C., Xiong, K., Mirghassemi, F., & Tran, H. (2017). Attention training to pleasant stimuli in anxiety. Biological Psychology, 122, 80-92.
20. Short, N. A., & Schmidt, N. B. (2020). Developing and testing a novel, computerized insomnia and anxiety intervention to reduce safety aids among an at-risk student sample: A randomized controlled trial. Behavior Therapy, 51(1), 149-161.
21. Skodzik, T., Adelt, M. H., Nossek, V. A., Kuck, S. T., & Ehring, T. (2018). Does a novel training in mental imagery reduce pathological worry?. Behaviour Research and Therapy, 109, 56-67.
22. Teng, M.-H., Hou, Y.-M., Chang, S.-H., & Cheng, H.-J. (2019). Home-delivered attention bias modification training via smartphone to improve attention control in sub-clinical generalized anxiety disorder: A randomized, controlled multi-session experiment. Journal of Affective Disorders, 246, 444-451.
23. Topper, M., Emmelkamp, P. M., Watkins, E., & Ehring, T. (2017). Prevention of anxiety disorders and depression by targeting excessive worry and rumination in adolescents and young adults: A randomized controlled trial. Behaviour research and therapy, 90, 123-136.
24. Vrijsen, J. N., Dainer-Best, J., Witcraft, S. M., Papini, S., Hertel, P., Beevers, C. G., ... & Smits, J. A. (2019). Effect of cognitive bias modification-memory on depressive symptoms and autobiographical memory bias: Two independent studies in high-ruminating and dysphoric samples. Cognition and Emotion, 33(2), 288-304.
25. Wilkinson, P. O., & Goodyer, I. M. (2008). The effects of cognitive-behavioural therapy on mood-related ruminative response style in depressed adolescents. Child and Adolescent Psychiatry and Mental Health, 2(1), 1-10.
26. Wong, D. F. K., Ng, T. K., Zhuang, X. Y., Wong, P. W., Leung, J. T., Cheung, I. K. M., & Kendall, P. C. (2020). Cognitive-behavior therapy with and without parental involvement for anxious Chinese adolescents: A randomized controlled trial. Journal of Family Psychology, 34(3), 353.
27. Yang, W., Zhang, J. X., Ding, Z., & Xiao, L. (2016). Attention bias modification treatment for adolescents with major depression: A randomized controlled trial. Journal of the American Academy of Child & Adolescent Psychiatry, 55(3), 208-218.
28. Zemestani, M., Davoodi, I., Honarmand, M. M., Zargar, Y., & Ottaviani, C. (2016). Comparative effects of group metacognitive therapy versus behavioural activation in moderately depressed students. Journal of Mental Health, 25(6), 479-485.

**Supplementary 5.** Funnel plot of standard error by Hedges' g in observed and imputed studies examining the effect of active treatment compared with control conditions on RNT, depression and anxiety outcomes at post-test


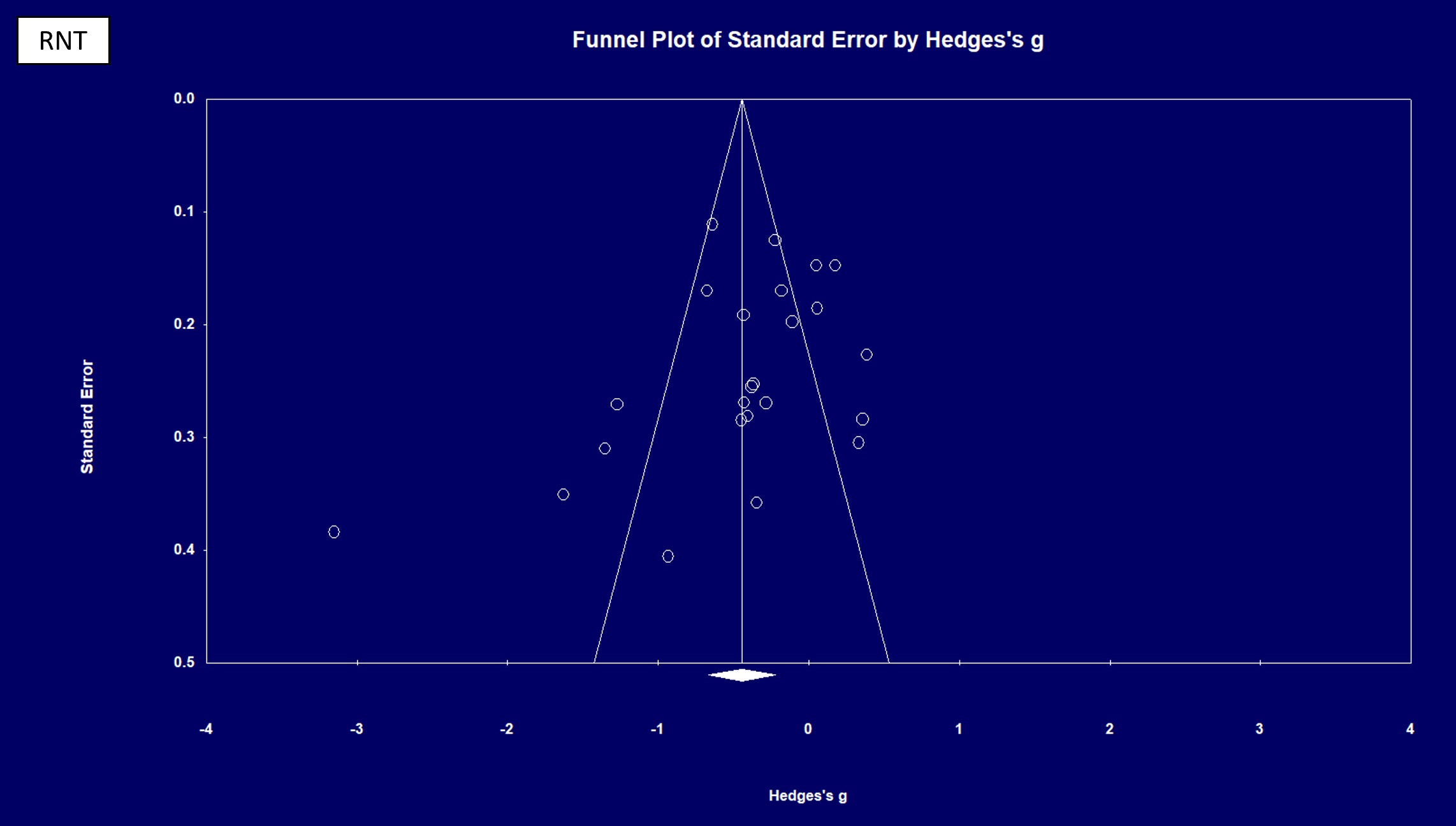


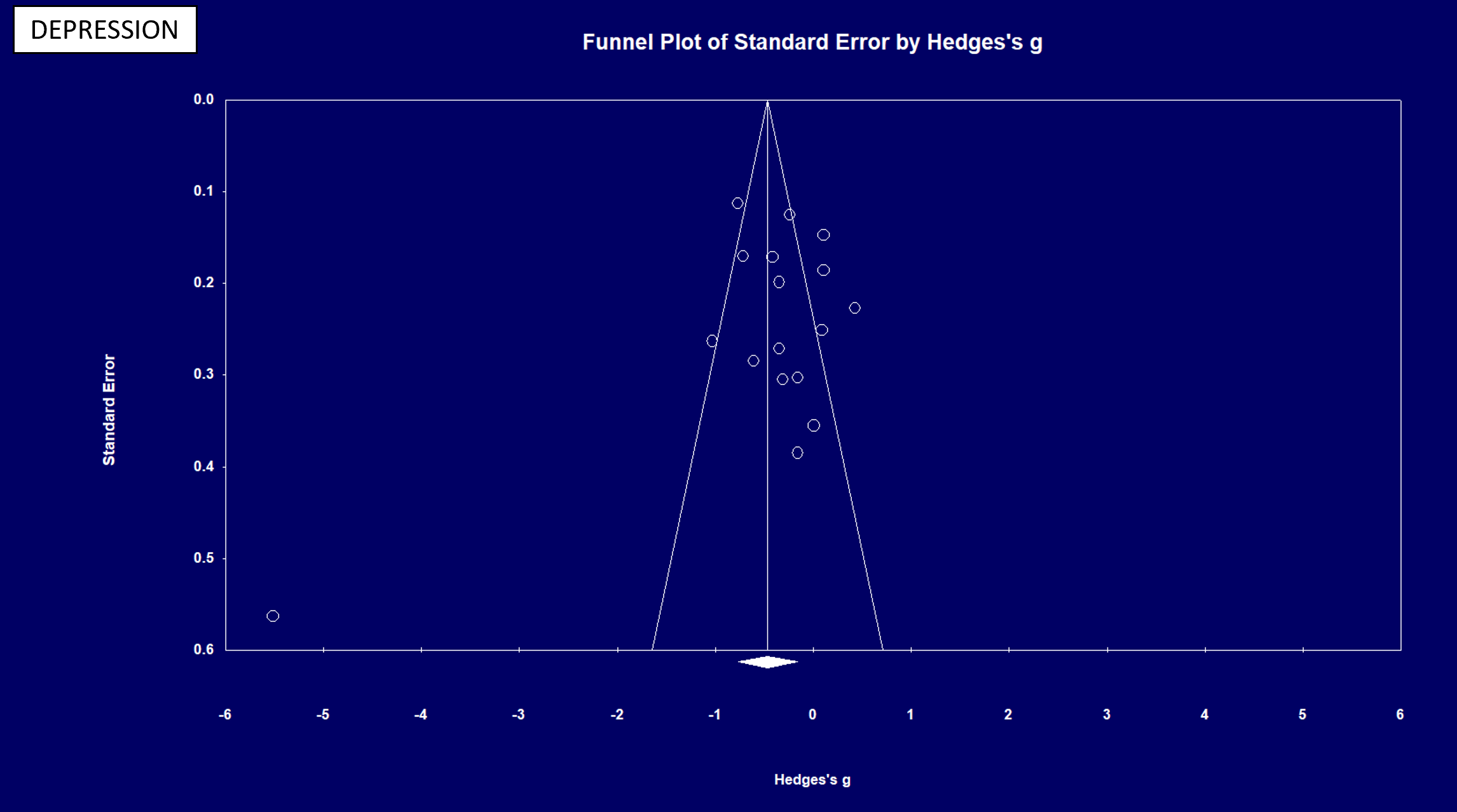


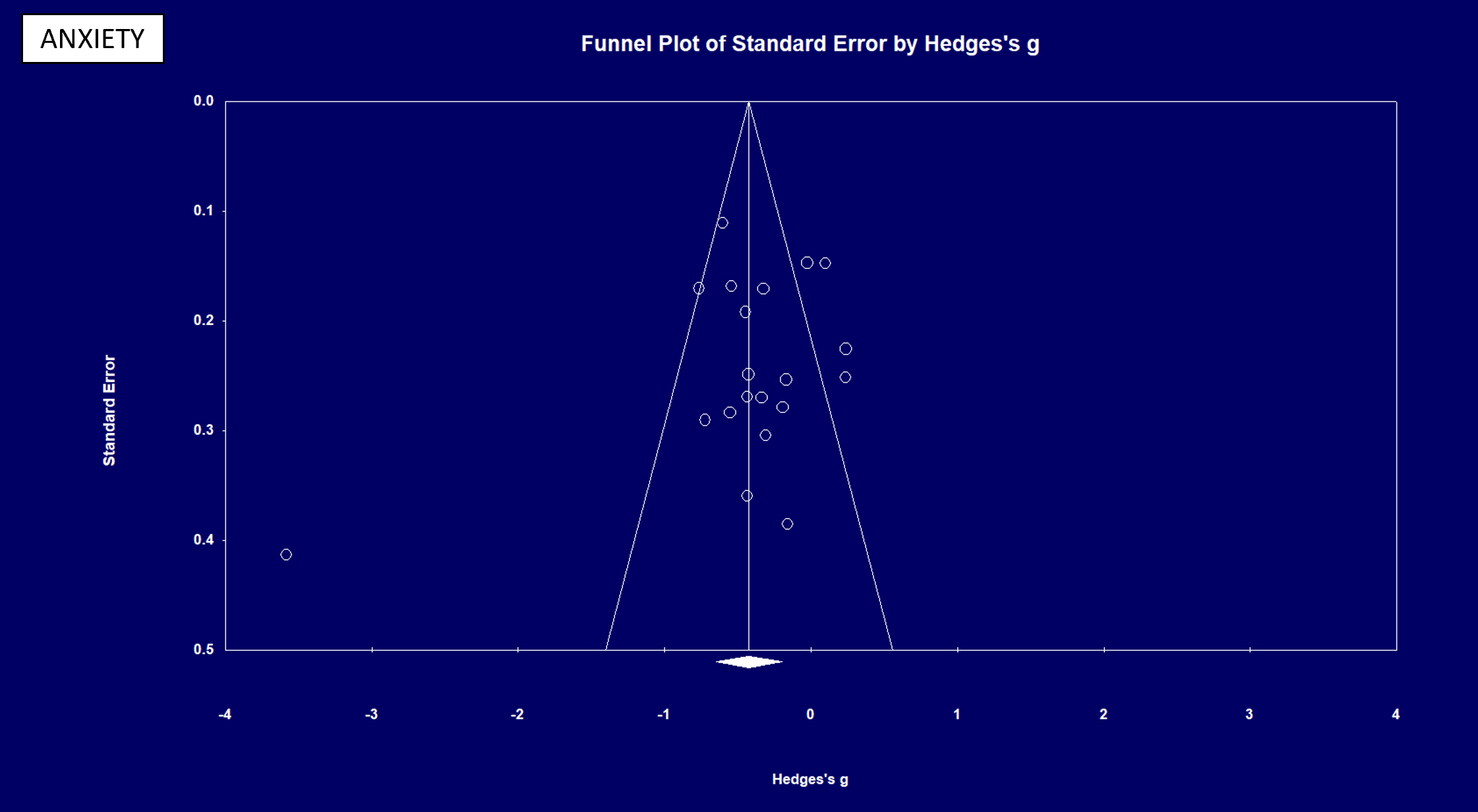


**Supplementary 4.** Meta regression results with outlier trial (Zemestani et al., 2016) included

| Relationship with RNT | | Slope coefficient | Standard error (95% CI) | Z-value | *p-*value |
| --- | --- | --- | --- | --- | --- |
| RNT focused interventions | |  |  |  |  |
|  | Depression outcomes | 0.65 | 0.13 (0.37: 0.92) | 4.66 | <.001 |
|  | Anxiety outcomes | 0.89 | 0.19 (0.50: 1.27) | 4.55 | <.001 |
| Non-RNT focused interventions | |  |  |  |  |
|  | Depression outcomes | 0.42 | 0.13 (0.18: 0.67) | 3.36 | <.01 |
|  | Anxiety outcomes | 0.70 | 0.25 (0.20: 1.19) | 2.74 | <.05 |

**Supplementary 5.** Risk of bias rating for each trial

|  | **Randomization process** | **Deviations from intended interventions** | **Missing outcome data** | **Measurement of the outcome** | **Selection of the reported result** | **Overall rating** |
| --- | --- | --- | --- | --- | --- | --- |
| Bernal-Manrique et al 2020 | (+) | (-) | (+) | (+) | (+) | Low |
| De Voogd De Hullu et al 2017 | (+) | (+) | (+) | (+) | (+) | Low |
| De Voogd Wiers 2017 | (+) | (+) | (+) | (+) | (-) | Low |
| Grol et al 2018 | (+) | (+) | (+) | (+) | (-) | Low |
| Idsoe et al 2019 | (+) | (+) | (-) | (-) | (-) | High |
| Kauer et al 2012 | (+) | (+) | (+) | (+) | (-) | Low |
| Kocovski et al 2019 | (-) | (+) | (-) | (+) | (-) | High |
| LaFreniere et al 2016 | (+) | (+) | (+) | (+) | (-) | Low |
| Lytle et al 2002 | (+) | (+) | (+) | (+) | (-) | Low |
| McDermott et al 2021 | (+) | (+) | (+) | (+) | (-) | Low |
| McEvoy et al 2017 | (+) | (+) | (+) | (+) | (-) | Low |
| McIndoo et al 2016 | (+) | (+) | (+) | (+) | (-) | Low |
| McIntosh et al 2013 | (-)(-) | (-) | (+) | (+) | (-) | High |
| Modini et al 2017 | (+) | (+) | (+) | (+) | (-) | Low |
| Modini et al 2018 | (+) | (+) | (+) | (+) | Low | Low |
| Mogoaşe et al 2013 | (+) | (+) | (+) | (+) | (-) | Low |
| Norr et al 2014 | (-) | (+) | (+) | (+) | (-) | High |
| Richards et al 2016 | (+) | (+) | (+) | (+) | (-) | Low |
| Sass et al 2017 | (-) | (+) | (+) | (+) | (-) | High |
| Short et al 2020 | (-) | (+) | (-) | (+) | (-) | High |
| Skodzik et al 2018 | (-) | (+) | (+) | (+) | (-) | High |
| Teng et al 2019 | (+) | (+) | (-) | (+) | (-) | High |
| Topper et al 2017 | (+) | (+) | (+) | (+) | (-) | Low |
| Vrijsen et al 2019 | (-) | (+) | (-)(-) | (+) | (-) | High |
| Wilkinson et al 2008 | (-) | (+) | (+) | (+) | (-) | Low |
| Wong et al 2020 | (+) | (+) | (+) | (+) | (-) | Low |
| Yang et al 2015 | (+) | (+) | (+) | (+) | (-) | Low |
| Zemestani et al 2016 | (-) | (+) | (+) | (+) | (-) | High |

**Supplementary 6.** Sensitivity analyses of subgroup comparisons between process and content focused interventions re-coding attention bias modification

|  | | Number of studies | Hedge’s *g* (95% CI) | *p-*value | Heterogeneity (I^2^) |
| --- | --- | --- | --- | --- | --- |
| **Subgroups** | | | | | |
| Content v process ORIGINAL | | | | | |
|  | Content focused intervention | 14 | -0.13 (-0.28: 0.01) | .01 | 38.28 |
|  | Process focused intervention | 10 | -0.85 (-1.29: -0.41) |  | 89.45 |
| Content v process SENSITIVITY ANALYSIS | | | | | |
|  | Content focused intervention | 9 | -0.20 (-0.35: -0.04) | .05 | 19.85 |
|  | Process focused intervention | 15 | -0.56 (-0.91: -0.22) |  | 89.45 |
